# Schizophrenia spectrum disorders following past exposure to ionizing radiation and SARS CoV-2 infection

**DOI:** 10.1192/j.eurpsy.2022.1226

**Published:** 2022-09-01

**Authors:** K. Loganovsky, T. Loganovskaja

**Affiliations:** State Institution “National Research Centre for Radiation Medicine of National Academy of Medical Sciences of Ukraine”, Radiation Psychoneurology, Institute Of Clinical Radiology, Kyiv, Ukraine

**Keywords:** Schizophrenia spectrum disorders, Ionizing radiation, COVID-19 pandemiс, SARS CoV-2 infection

## Abstract

**Introduction:**

Whether exist a potential association between schizophrenia spectrum disorders following past exposure to ionizing radiation and SARS CoV-2 infection is unknown.

**Objectives:**

To assess a possible role of double radiation-viral exposure in pre- and postnatal periods in schizophrenia spectrum disorders genesis.

**Methods:**

Integration and analysis of information available with the results of own clinical and epidemiological studies.

**Results:**

The renaissance of interest to the viral hypothesis of schizophrenia is observing during the current COVID-19 pandemic. There is an increasing number of cases and case series reports on psychotic schizophreniform disorders following SARS CoV-2 infection diagnosed as COVID-19-asssociated brief psychotic disorder, first episode psychosis, acute and transient psychotic disorder. The prevalence rate of schizophrenia in A-bomb survivors in Nagasaki was very high – 6 % (Nakane and Ohta, 1986), and increased in those prenatally exposed to A-bombing (Imamura et al., 1995) and medical X-irradiation (Gross et al., 2018). We found a significant increase in the schizophrenia incidence in the Chornobyl exclusion zone personnel, as well as schizophreniform syndromes in Chornobyl clean-up workers (liquidators) irradiated by moderate to high doses (more than 0.30 Sv). The neural diathesis-stressor hypothesis of schizophrenia spectrum disorders was proposed (Loganovsky and Loganovskaja, 2000; Loganovsky et al., 2005). Recently we observed the clinical case of organic schizophrenia-like disorder in the liquidator who was ill with COVID-19.

**Conclusions:**

The linkage between schizophrenia spectrum disorders following past exposure to ionizing radiation and SARS CoV-2 infection can exist that should be studied on the irradiated cohorts with following COVID-19.

**Disclosure:**

No significant relationships.

